# Effect of gait training using Welwalk on gait pattern in individuals with hemiparetic stroke: a cross-sectional study

**DOI:** 10.3389/fnbot.2023.1151623

**Published:** 2023-04-17

**Authors:** Takuma Ii, Satoshi Hirano, Daisuke Imoto, Yohei Otaka

**Affiliations:** ^1^Faculty of Rehabilitation, School of Health Sciences, Fujita Health University, Toyoake, Aichi, Japan; ^2^Department of Rehabilitation Medicine I, School of Medicine, Fujita Health University, Toyoake, Aichi, Japan; ^3^Department of Rehabilitation, Fujita Health University Hospital, Toyoake, Aichi, Japan

**Keywords:** gait training, hemiparesis, rehabilitation, RAGT, robot, stroke

## Abstract

**Introduction:**

We aimed to explore the effect of gait training using Welwalk on gait patterns by comparing differences in gait patterns between robotic-assisted gait training using Welwalk and gait training using an orthosis in individuals with hemiparetic stroke.

**Methods:**

This study included 23 individuals with hemiparetic stroke who underwent gait training with Welwalk combined with overground gait training using an orthosis. Three-dimensional motion analysis on a treadmill was performed under two conditions for each participant: during gait training with Welwalk and with the ankle-foot orthosis. The spatiotemporal parameters and gait patterns were compared between the two conditions.

**Results:**

The affected step length was significantly longer, the step width was significantly wider, and the affected single support phase ratio was significantly higher in the Welwalk condition than in the orthosis condition. The index values of abnormal gait patterns were significantly lower while using Welwalk than in the orthosis condition. The following four indices were lower in the Welwalk condition: contralateral vaulting, insufficient knee flexion, excessive hip external rotation during the paretic swing phase, and paretic forefoot contact.

**Discussion:**

Gait training using Welwalk increased the affected step length, step width, and single support phase while suppressing abnormal gait patterns as compared to gait training using the ankle-foot orthosis. This study suggests that gait training using Welwalk may promote a more efficient gait pattern reacquisition that suppresses abnormal gait patterns.

**Trial registration:**

Prospectively registered in the Japan Registry of Clinical Trials (https://jrct.niph.go.jp; jRCTs042180152).

## 1. Introduction

Gait disorders after stroke are commonly faced by patients. Many people with severe motor paralysis after a stroke require assistance while walking (Wade et al., 1985). Although they achieve independent walking, the risk of falling remains owing to walking instability, and decreased walking speed limits activities of daily living (Perry et al., 1995; Blennerhassett et al., 2012). Dysfunction of the paralyzed leg (Dean and Kautz, 2015) and lack of balance function (Lorenze et al., 1958; Chen et al., 2005) have been reported as causes of gait instability in patients with post-stroke hemiparesis. Spatiotemporal parameters, such as decreased stride length and shortened single support phase ratio of the affected side during walking, are altered in individuals with hemiparetic stroke compared to those with a normal gait (Perry et al., 1995; Chen et al., 2005). Furthermore, individuals with hemiparetic stroke show compensatory movements due to decreased clearance caused by insufficient hip and knee flexion and ankle dorsiflexion (Lorenze et al., 1958; Woolley, 2001; Lamontagne et al., 2002; Chen et al., 2005). The abnormal gait pattern of individuals with hemiparetic stroke is not determined solely by the recovery of motor paralysis, but rather, it is strategically learned during the gait training process (Huitema et al., 2004). Therefore, the acquired gait pattern depends on the content of the walking training. The search for the optimal method of gait training is an important issue.

Several methods of gait training have been proposed for individuals with post-stroke hemiparesis, including high-speed waking training speed and partial body weight support treadmill training with functional electrical stimulation (Pohl et al., 2002: Wang et al., 2022). One of the standard approaches to gait training in individuals with hemiparetic stroke—lower-limb orthosis—is used to improve gait patterns in patients with hemiparesis after stroke at any stage of gait training (Veerbeek et al., 2014; Johnston et al., 2021). Furthermore, its efficacy has been verified (Woolley, 2001; Tyson et al., 2013). Gait training using an ankle-foot orthosis (AFO) in individuals with hemiparetic stroke increases the gait speed, stride length, and ratio of the affected single-leg support phase (Danielsson and Sunnerhagen, 2004; Thijssen et al., 2007; Nikamp et al., 2017). Furthermore, it increases clearance during the swing phase by maintaining the dorsiflexion angle of the ankle joint and improving the hip and knee angles during the stance phase (Tyson et al., 2013; Nikamp et al., 2017). Lately, robots have been used for gait training during rehabilitation. The efficacy of robot-assisted gait training (RAGT) has been reported (Mehrholz et al., 2020), which increases gait independence and gait speed, and changes the spatiotemporal parameters of gait (Hidler et al., 2009; Westlake and Patten, 2009). Individuals with hemiparesis who received conventional gait training combined with RAGT exhibited a less compensatory gait pattern at discharge compared to conventional gait training alone in a matched control (Katoh et al., 2020).

However, most of these studies compared different individuals. Thus, the changes in gait pattern observed in RAGT compared to using an orthosis simultaneously in the same individuals with post-stroke hemiparesis at the same time are unclear. Clarifying the differences in gait characteristics influenced by training conditions of the same individual is important to assess the mechanism of efficacy of RAGT.

This prospective study explained the differences in gait patterns between two conditions: robot-assisted gait training and gait training using an orthosis in individuals with post-stroke hemiparesis. We hypothesized that RAGT elicits a spatiotemporal parameter closer to normal and a gait pattern with suppressed compensatory movement than when using an orthosis, thanks to robotic assistance.

## 2. Materials and methods

### 2.1. Study design and setting

This prospective study was approved by the Fujita Health University Ethics Review Committee (CR22-003) and all participants provided written consent before the study. This study included 23 individuals with post-stroke hemiparesis admitted to Fujita Health University Hospital who met the criteria for the use of Welwalk (Supplementary Table 1) and underwent three-dimensional gait analyses on a treadmill under two conditions (with Welwalk and with an AFO) within 1 week. Specifically, from April 2018 to March 2021, 80 individuals met the criteria for the use of Welwalk; 23 of whom were able to complete a gait analysis for this study. This study compared the gait patterns of the participants under the two conditions cross-sectionally.

### 2.2. Participants

The participants were given a combination of gait training using a lower-limb orthosis and RAGT with Welwalk. The physician and therapist determined the start time of RAGT, starting as early as possible after the onset of the stroke. The frequency of the RAGT was 5–6 days a week for 40 min per day. The primary target of gait training using Welwalk was to improve gait independence. Therefore, based on the idea of assist-as-needed (Srivastava et al., 2015), during the training, the parameters of assistance function of Welwalk were adjusted to minimize therapist assistance, followed by minimizing robot assistance. For example, the knee extension assist level was adjusted according to each patient's risk of falling, increased if excessive knee flexion occurred frequently during the stance phase, and decreased if sufficient knee extension was consistently obtained. In addition to RAGT, the participants received rehabilitation, including physical therapy, occupational therapy, and speech-language therapy, if needed, 6–7 days a week. In total, including RAGT, the participants underwent rehabilitation for 3 h per day. Physical therapy interventions other than RAGT were not controlled, and the therapist was in charge of each participant performing walking and standing training using orthotics.

### 2.3. Robotic apparatus used

**Figure 1:**
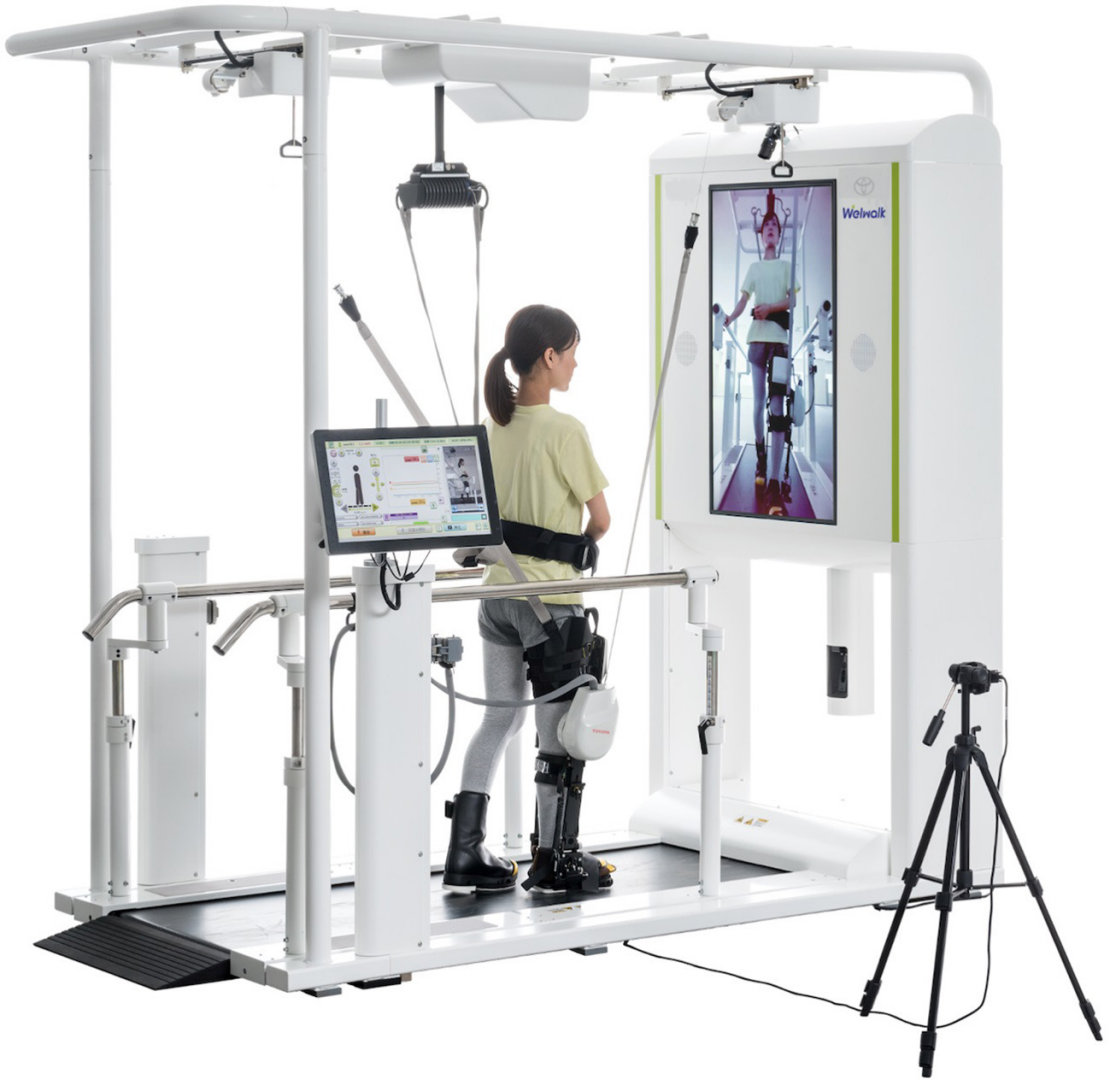
Welwalk WW-2000. The figure is published with consent from Toyota Motor Corporation.

Welwalk (WW-2000, WW-1000, Toyota Motor Corporation, Japan) (Figure 1) was used for the RAGT (Hirano et al., 2017). Welwalk is a walking exercise assistance system in which a knee-ankle-foot orthosis-type robot is attached to a paralyzed leg and the patient walks on a treadmill using a safety suspension. An improvement in walking independence when using Welwalk for individuals with post-stroke hemiparesis has been reported (Tomida et al., 2019; Ii et al., 2020). The robot leg can assist in supporting knee extension with ten levels of assistance during the stance phase (knee extension assist). The maximum assistance level can hold the participant's knee in the extension position preventing knee bending during normal walking (assist level 10: the target torque is approximately 80 Nm). The minimum assistance level does not assist knee extension during the stance phase (assist level 1: the target torque is approximately 2 Nm). During the swing phase, the wire pulled the leg of the robot (swing assist). The traction force can be adjusted to six levels in the vertical direction and three levels in the anteroposterior direction. The left and right positions of the swing assist can be adjusted in four steps, allowing adjustment of adduction/abduction and internal/external rotation of the hip joint during the affected swing phase. The vertical assistance force is the same at level 2 as the weight of the robot's leg, and changes by 0.5 kg for every 1-level change. In addition, the robotic knee joint flexed and extended during the swing phase to easily swing the paralyzed leg. The flexion angle, time, and timing of the beginning of knee flexion were also adjusted. The ankle joint of the robotic leg can be set to a limited angle of plantar dorsiflexion like an orthosis. As a visual feedback item for participants, the front monitor can be switched between a mirror-like image, an image from the sagittal plane, and an image showing only the feet to improve gait posture and pattern. The treadmill could be adjusted from 0.2–3.0 km/h. During training, the therapist stood behind the participants and adjusted the settings according to their specific gait pattern (Hirano et al., 2017).

### 2.4. Gait analysis

The primary outcome was the change in spatio-temporal parameters during walking on the treadmill. The secondary outcomes were the index values of 12 abnormal gait patterns that typically appear in individuals with post-stroke hemiparesis (Table 1) (Itoh et al., 2012; Tanikawa et al., 2016, 2021; Hishikawa et al., 2018). Analyses were performed when participants were able to walk without the help of a therapist during the gait training period in both training conditions: with Welwalk and with an AFO. The order of gait analysis was not specific. To avoid the influence of fatigue, gait analyses were not conducted consecutively for both conditions. The types of orthoses used were determined by the physician and the therapist in charge.

**Table 1:** Definitions of index values representing the severity of abnormal gait.

**Abnormal gait**	**Definition**
Circumduction gait	The lower extremity of the affected side shows hip joint abduction and lateral rotation during the initial swing to mid-swing and hip joint adduction and medial rotation during mid-swing to terminal swing, following a semicircular trajectory.
Hip hiking	The pelvis on the affected side is raised during pre-swing to mid-swing, associated with the shortening of the trunk on the affected side.
Retropulsion of the hip	The affected hip joint does not move continually forward over the affected ankle joint during loading response to mid-stance.
Excessive hip external rotation	A more excessive hip external rotation of the affected leg than normal throughout the entire swing phase.
Knee extensor thrust	A dynamic, rapid knee extension during the loading response to terminal stance on the affected leg.
Flexed-knee gait	The knee remains in a flexed position throughout the stance phase.
Insufficient knee flexion during the swing phase	A decrease in the maximum knee flexion angle during the swing phase.
Forefoot contact	At initial contact, the forefoot on the affected side is the first to make contact, regardless of walking brace use.
Medial whip	Medial rotation of the ankle around the toe as a pivot during terminal stance to pre-swing.
Excessive lateral shift of the trunk over the unaffected side	The trunk shifts excessively over the unaffected side during the swing phase of the affected side.
Contralateral vaulting	Excessive pelvic rise by the unaffected side knee extension during the stance phase of the unaffected side.
Posterior pelvic tilt	Posterior tilting of the pelvis in the sagittal plane is used to swing the leg during the swing phase.

A three-dimensional motion analysis system (Kinema Tracer, KISSEICOMTEC, Matsumoto, Japan) was used for the measurements. In the three-dimensional motion analysis, color markers were attached bilaterally to the shoulder (acromion), hip joint (at one-third of the distance from the great trochanter on a line connecting the anterior superior iliac spine and the great trochanter), knee joint (at the mid-point of the anteroposterior diameter of the lateral femoral epicondyle), ankle joint (at the lateral malleolus), and toes (at the heads of the fifth metatarsal) at 12 locations in the orthosis condition. In the Welwalk condition, two of the marker positions in the orthosis condition, the knee joint and ankle joint centers, were affixed to the robot leg. The distance to the actual knee joint and ankle joint position from the marker was measured and corrected to calculate and analyze the same joint positions as in the orthosis condition.

Each measurement was performed for 20 seconds at a sampling frequency of 60 Hz. In the orthosis condition, measurements were performed on the treadmill at a comfortable walking velocity for level walking or at 70% of this comfortable walking velocity if the patient had difficulty walking at that velocity. Measurements in the Welwalk condition were performed at a comfortable walking velocity on Welwalk and optimal assistance settings. In both conditions, all participants walked while viewing their walking posture on a monitor in front of them, and they used a handrail during the measurement. Additionally, the values of the robot's swing assist and knee extension assist were recorded in the Welwalk condition.

### 2.5. Statistical analysis

First, spatiotemporal parameters, including walking velocity, cadence, stride length, step length, and step width, were compared between the Welwalk and orthosis conditions. Second, the crude time and percentage of each phase of the gait cycle were compared between the two conditions. Each gait cycle was divided into four phases: affected initial double support, affected single support, unaffected initial double support, and affected swing. Finally, the 12 indices of abnormal gait patterns between the two conditions were compared.

Comparisons were performed using a paired *t-*test or Wilcoxon signed-rank test, according to the type of variable. Statistical analyses were performed using SPSS version 21 software (IBM Corp., Armonk, NY, USA). *P*-values < 0.05 were considered statistically significant.

## 3. Results

Participant demographic data are presented in Table 2. The median Stroke Impairment Assessment Set lower extremity motor function total score (hip-flexion test, knee-extension test, foot-pat test) as the degree of paralysis was 5 (4–6), indicating moderate motor paralysis (Chino et al., 1994). The robot assists in the Welwalk condition ranged from 1 to 10 (median, 6) for the swing assist and from 1 to 6 (median, 3) for the knee extension assist. In the orthosis condition, all participants used the Remodeled Adjustable Posterior Strut (RAPS)-AFO (Mizuno et al., 2005).

**Table 2:** Participant characteristics (*n* = 23).

**Variables**	
Age, mean (SD), year	60.1 (11.2)
Types of strokes, ischemic/hemorrhage, *n*	3/20
Hemiparetic side, right/left, *n*	12/11
Days after stroke onset, mean (SD)	
At the measurement with the Welwalk	53 (27)
At the measurement with an AFO	53 (28)
FIM-walk score, median (IQR)	5 (5–5)
SIAS- motor L/E score, median (IQR)	5 (4–6)
Sex, male/female, *n*	16/7

SD, standard deviation; IQR, interquartile range; AFO, ankle-foot orthosis; FIM, functional independence measure; SIAS, Stroke Impairment Assessment Set.

A comparison of the spatiotemporal parameters between the two conditions is presented in Table 3. Walking velocity and cadence showed no significant difference between the two conditions (*P* = 0.485 and *P* = 0.130, respectively). Stride length and affected step length were significantly longer in the Welwalk condition than in the orthosis condition (*P* < 0.00 and *P* = 0.001, respectively). The width of the steps was significantly wider in the Welwalk condition than in the orthosis condition (*P* < 0.001).

**Table 3:** Spatiotemporal parameters of gait.

	**Welwalk**	**AFO**	***P*-values**
Velocity, mean (SD), km/h	0.7 (0.2)	0.7 (0.4)	0.485
Cadence, mean (SD), steps/min	56.0 (0.8)	60.5 (17.1)	0.130
Stride length, mean (SD), cm	44.5 (13.2)	37.2 (13.1)	< 0.001
Step length, mean (SD), cm			
Affected side	26.4 (6.3)	20.7 (7.1)	0.001
Unaffected side	18.1 (10.2)	16.5 (9.5)	0.331
Step width, mean (SD), cm	20.2 (2.8)	14.7 (2.8)	< 0.001

SD, standard deviation; AFO, ankle-foot orthosis.

Comparisons of gait cycles are presented in Table 4. The percentage of affected single supports was significantly higher in the Welwalk condition than in the orthosis condition (*P* = 0.016). There were no significant differences in other parameters (Table 4).

**Table 4:** Temporal parameters of the gait cycle.

	**Time, s**			**Ratio, %**		
	**Welwalk**	**AFO**	***P*** **-values**	**Welwalk**	**AFO**	***P*** **-values**
Affected initial double support	0.47 (0.14)	0.50 (0.23)	0.525	21.3 (6.2)	23.1 (5.3)	0.296
Affected single support	0.41 (0.19)	0.31 (0.09)	0.003	18.3 (6.2)	15.5 (5.3)	0.016
Unaffected initial double support	0.64 (0.17)	0.69 (0.39)	0.500	29.2 (6.6)	30.5 (9.0)	0.499
Affected swing	0.69 (0.20)	0.66 (0.23)	0.565	31.2 (6.6)	30.1 (6.4)	0.913

Values are presented as mean (standard deviation). AFO, ankle-foot orthosis.

**Figure 2:**
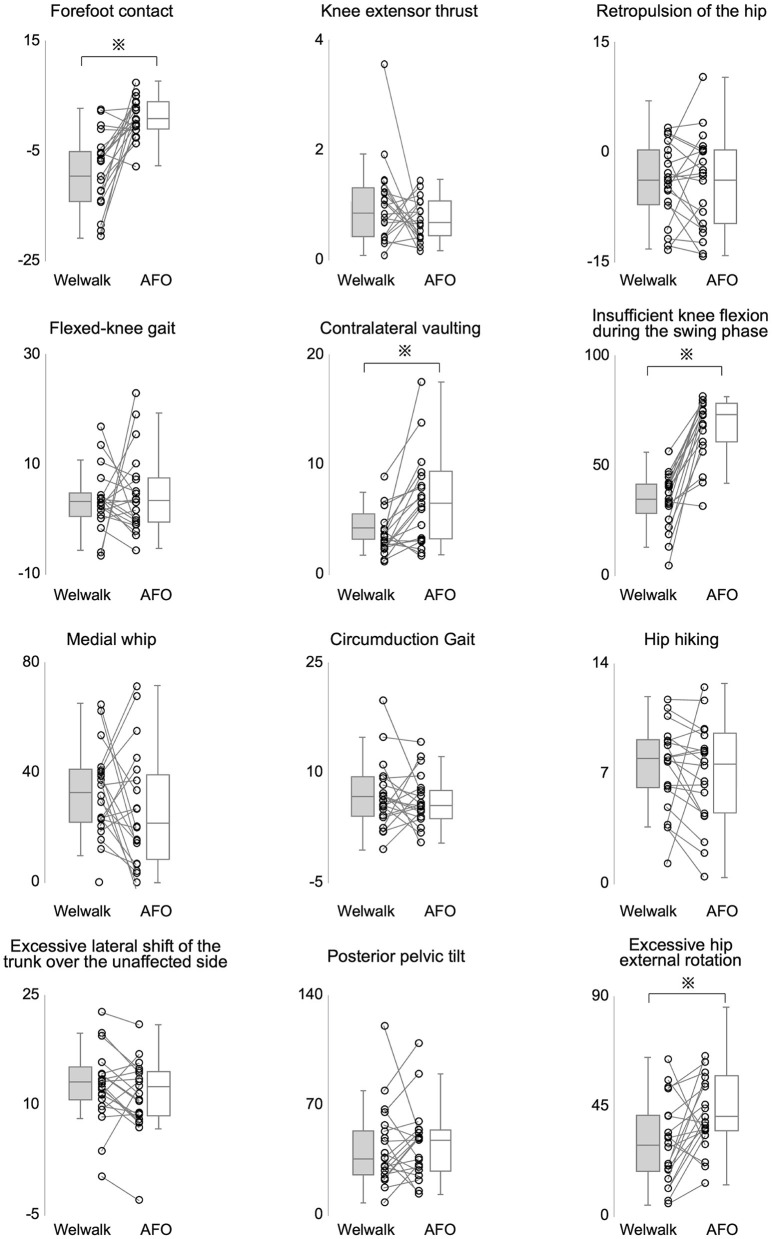
Comparison of the severities of abnormal gait patterns. A comparison of index values representing the severity of abnormal gait patterns between the Welwalk and the orthosis conditions is shown. The vertical axis represents the index values. All index values were compared using the Wilcoxon-signed rank test. ^*^P-values < 0.05 were considered statistically significant.

Of the 12 index characteristics of hemiparetic stroke calculated from the three-dimensional motion analysis, the values of forefoot contact, contralateral vaulting, insufficient knee flexion during the swing phase, and excessive hip external rotation were lower in the Welwalk condition than in the orthosis condition (*P* < 0.001, *P* = 0.001, *P* < 0.001, and *P* = 0.011, respectively). There were no significant differences in the other eight indices between the two conditions (Figure 2).

## 4. Discussion

This study investigated the effects of Welwalk use on gait patterns in individuals with post-stroke hemiparesis by comparing the gait patterns between two conditions: robotic-assisted gait training using Welwalk and using an orthosis. The present results showed that gait training using Welwalk can increase the affected step length, step width, and single support phase time by suppressing abnormal gait patterns during gait compared to gait training using an orthosis.

Among the basic spatiotemporal parameters of walking, stride length and affected step length were significantly longer while using the Welwalk than the orthosis. The swing assist provided by Welwalk might have compensated for the dysfunction of the affected leg swing when the affected side had low swing ability due to paresis. The swing assist and knee flexion during swing were adjusted to ensure clearance and reduce the risk of falls; however, this might have also indirectly influenced stride extension.

Regarding step width, a previous study reported that the step width of Japanese healthy adults was 23.3 cm (Mukaino et al., 2016). The individuals with post-stroke hemiparesis who were able to walk independently displayed wider step widths compared to healthy individuals, reported to be 28.2 cm (Mukaino et al., 2016) and 29.8 cm (Abe et al., 2009). The participants in our study showed a narrower step width in both conditions (Welwalk and orthosis) than those previously reported in healthy individuals (Mukaino et al., 2016). This finding may be attributed to the different conditions between the present study and previous studies (Abe et al., 2009; Mukaino et al., 2016) regarding the time after stroke onset, walking speed, and use of a handrail. In a longitudinal study of gait parameters in hemiparetic stroke, no change in step width was observed after stroke onset (Chow and Stokic, 2021). It was also reported that changing walking speed did not change the step width. Therefore, we consider that the effect of handrail use contributed to this result (Kao et al., 2014). All participants in our study used a handrail, while participants in previous studies did not. Handrails have been reported to reduce step width by approximately 25% (IJmker et al., 2015). Welwalk showed a wider step width when compared to the orthosis. This difference is thought to be due to the adjustability of the step width with Welwalk. The adjustment functions of Welwalk enable the therapist to adjust the left-right position of the paralyzed side swing by changing the position of swing assist and to prompt the patient to position the paralyzed foot contact on the monitor for visual feedback (Hirano et al., 2017). Individuals with stroke are considered to widen their step width to increase walking stability (Hak et al., 2013), and training with a wider step width leads to increased walking stability (McAndrew Young and Dingwell, 2012). This may contribute to the faster independence of walking achieved in RAGT with Welwalk than in walking training with an orthosis (Ii et al., 2020).

Regarding the temporal parameters of the gait cycle, the percentage of affected single support phase was significantly larger in the Welwalk condition than in the orthosis condition. This suggests that the function of the knee extension assist of the Welwalk is useful for individuals with post-stroke hemiparesis when the use of an AFO is insufficient to provide stability in the stance phase. Individuals with post-stroke hemiparesis had been reported to tend to shorten the affected single support phase ratio due to decreased stance support and balance on the paralyzed support (Goldie et al., 2001). Furthermore, the single-support phase ratio of the affected side increased as gait training progressed and was associated with increased walking speed in individuals with post-stroke hemiparesis (De Quervain et al., 1996; Goldie et al., 2001). In RAGT with Welwalk, the participants could walk at a gait cycle similar to that of the later stage when training had progressed, and their gait ability had improved.

Regarding gait patterns, four indices (insufficient knee flexion, forefoot contact, excessive hip external rotation, and contralateral vaulting during the paretic swing phase) were significantly lower in the Welwalk condition than in the orthosis condition. Because this index value represents the degree of abnormal gait pattern, the lower the value, the closer it is to a normal gait pattern. Thus, the Welwalk condition elicits a gait pattern closer to normal, suggesting that the patient can walk with a suppressed abnormal gait pattern. Insufficient knee flexion during the swing phase is caused by decreased hip flexor output and spasticity of the rectus femoris muscle (Perry et al., 2010) and is considered a factor leading to decreased toe clearance (Gage et al., 1987). In the Welwalk condition, the direct knee joint flexion motion of the robot during the swing phase could maintain clearance and reduce abnormal gait patterns. Forefoot contact is caused by insufficient knee extension before initial contact, reduced ankle dorsiflexor output, and spasticity of the plantar flexor muscle (Perry et al., 2010). Therefore, it is believed that the lack of knee extension prior to initial contact was compensated for by knee extension in the terminal swing by Welwalk, leading to a reduction in forefoot contact. Excessive hip external rotation is determined by the direction of the foot during the swing leg (Tanikawa et al., 2016), and is thought to be caused by reduced hip flexor output (Lorenze et al., 1958). In the Welwalk condition, the swing assists and knee flexion exerted by the robotic assist allowed for an easier paretic leg swinging, which indirectly reduced external rotation of the hip during the swing phase. Contralateral vaulting during the paretic swing phase is a compensatory movement for decreased paretic toe clearance owing to the output of the affected hip flexor (Woolley, 2001; Cruz and Dhaher, 2009; Matsuda et al., 2017). In the Welwalk condition, this compensatory movement might have been indirectly decreased by the robot's swing assist and knee flexion movements to maintain toe clearance.

The present study showed that RAGT using Welwalk increased the step length of the affected side and step width, and affected the single support phase while suppressing abnormal gait patterns during gait compared to gait training using the AFO. A longer step length is associated with greater joint moments of hip extension than a narrow step length and greater output of the gluteus maximus, gluteus medius, and other muscles involved in stance stability (Lim et al., 2017). Increasing the affected single support phase may also enhance the function of the proximal lower extremity (including the muscle around the hip), which contributes to the support of the stance phase (Gottschalk et al., 1989). RAGT using Welwalk may be more effective than gait training using AFO because it promotes increased muscle output around the hip joint, as it increases stride length and single support phase time during walking. The gait pattern during the training process is considered to lead to definitively acquired gait pattern. Changes in gait pattern specific to patients with hemiparesis can lead to gait instability (Dean and Kautz, 2015). Repeated gait training with suppressing abnormal gait patterns specific to hemiparesis is meaningful because it may eventually lead to improved gait stability and energy efficiency (Platts et al., 2006; Polese et al., 2018). RAGT combined with conventional rehabilitation in individuals with hemiparesis has previously been shown to improve independence while walking (Mehrholz et al., 2020; Moucheboeuf et al., 2020). Our study suggests that gait training with Welwalk may lead to a more efficient gait pattern with suppression of abnormal patterns in addition to an increased proportion of individuals with post-stroke hemiparesis who achieve gait independence.

The limitations of this study were that it was conducted at a single institution and only cross-sectionally. The training doses in the orthosis and Welwalk conditions before the measurement were not investigated. The multiple mechanisms of robotic assistance are associated with changes in various gait parameters, but their correspondence remains unclear. Furthermore, a longitudinal study with dose monitoring of each condition will elucidate the relationship between the type of training and the definitively acquired gait pattern.

## 5. Conclusion

We investigated the effect of RAGT using Welwalk on gait patterns by comparing gait patterns using Welwalk and AFO in individuals with post-stroke hemiparesis who received training for the use of Welwalk and an orthosis device. Compared to gait training using the AFO, RAGT using Welwalk can increase step length, step width, and single support phase time in the affected side while suppressing abnormal gait patterns.

## Data availability statement

The raw data supporting the conclusions of this article will be made available by the authors, without undue reservation.

## Ethics statement

The studies involving human participants were reviewed and approved by Fujita Health University Ethics Review Committee. The patients/participants provided their written informed consent to participate in this study.

## Author contributions

TI, SH, and YO designed this study, interpreted the data, and reviewed the manuscript. TI and DI collected and analyzed the data. TI and SH wrote the manuscript. All authors have read and approved the final manuscript.
