# Supplementary material for: Effect of gait training using Welwalk on gait pattern in individuals with hemiparetic stroke: a cross-sectional study
Source: Front Neurorobot. 2023 Apr 17;17:1151623. doi: 10.3389/fnbot.2023.1151623 (PMC10149761; doi:10.3389/fnbot.2023.1151623)
Supplement: Supplementary file 1 [file Table_1.DOCX]

Supplementary Material

Effect of gait training using Welwalk on gait pattern in individuals with hemiparetic stroke: a cross-sectional study

Takuma Ii^1^, Satoshi Hirano^2*^, Daisuke Imoto^3^, Yohei Otaka^2^

*** Correspondence:** Satoshi Hirano

E-mail: [sshirano@fujita-hu.ac.jp](mailto:sshirano@fujita-hu.ac.jp)

# Supplementary Table

**Supplementary Table 1.** Criteria for the use of Welwalk at Fujita Health University Hospital

| Inclusion criteria | Exclusion criteria |
| --- | --- |
| - Hemiparesis due to stroke | - Uncontrolled hypertension |
| - Within 90 days after stroke onset | - Restriction due to circulatory or respiratory problems |
| - Age: 20–85 years old | - Severe joint contractures or deformities |
| - SIAS lower extremity motor function score ≤ 12 | - Visual or auditory impairment that interfered with training |
| - FIM- walk ≤ 4 | - Pregnancy |
| - FIM- comprehension score ≥ 2 | - Possibility of incontinence contaminating the robot |
| - FIM- memory score ≥ 2 |  |
| - FIM- social interaction score ≥ 2 |  |
| - FIM- problem solving score ≥ 2 |  |

SIAS, Stroke Impairment Assessment Set; FIM, Functional Independence Measure.
